# The Applications of Decision-Level Data Fusion Techniques in the Field of Multiuser Detection for DS-UWB Systems

**DOI:** 10.3390/s151024771

**Published:** 2015-09-25

**Authors:** Yebo Gu, Minglei Yang, Zhenguo Shi, Zhilu Wu

**Affiliations:** 1 School of Electronics and Information Engineering, Harbin Institute of Technology, Harbin 150001, China; E-Mails: guyebo1987@gmail.com (Y.G.); shizhenguotvt@gmail.com (Z.S.); 2 Shanghai Electro-Mechanical Engineering Institute, Shanghai 200245, China; E-Mail: ahminglei1989@gmail.com

**Keywords:** data fusion, decision-level fusion criterion (DFC), multiuser detection (MUD), DS-UWB

## Abstract

In this paper, the decision-level data fusion techniques are extended to the multiuser detection (MUD) field. Then two novel MUD algorithms, that is the chairman arbitrating decision-level fusion criterion (CA-DFC) based MUD algorithm and the veto logic decision-level fusion criterion (VL-DFC) based MUD algorithm, are proposed for DS-UWB communication systems. In CA-DFC based method, the chairman can make his arbitration among the preliminary decisions from sub-optimal detectors by his own rule. In the VL-DFC based method, the undetermined bits in these preliminary decisions are considered to construct a simplified solution space, and then the chairman can make his final decision within this space. Simulation results demonstrate that the performances of CA-DFC and VL-DFC based MUD algorithms are superior to those of other sub-optimal MUD algorithms, and even close to that of OMD. Moreover, both of these proposed algorithms have lower computational complexity than OMD, which reveals their efficiency. Compared with CA-DFC, VL-DFC based algorithm achieves a little improvement in its performance, at the cost of the increment in its computational complexity. Thus, they can be applied to different practical situations.

## 1. Introduction

Data fusion is a significant technique for detection, estimation and decision making. As Joint Directors of Laboratories (JDL) defines [1], data fusion is a “multilevel, multifaceted process handling the automatic detection, association, correlation, estimation, and combination of data and information from several sources”. Hence, its objective is to make the optimal or sub-optimal use of the information generated by multiple knowledge sources and sensors. Now data fusion based systems are widely used in many areas, such as sensor networks, robotics, video and image processing, market planning, data mining, and knowledge discovery applications [2,3,4].

In the present literature, data fusion is usually categorized into three types: data-level, feature-level, and decision-level fusions [5,6]. To take account of the situations constrained on the communication bandwidth or the data storage capacity, the data-level and feature-level fusions may not always be applicable, compared with the decision-level fusion [5]. That is, preliminary decisions should be made at each local detector in order to compress the bits transmitted to the decision fusion center. Then this center will make a global decision based on these local decisions and some fusion criterions. Due to the various types of detector (or classifier, sensor) outputs, decision-level fusion considered in the literature can be divided into three categories [7]: the abstract level, the rank level, and the measurement level. In [8], the authors have classified many typical decision-level fusion methods into these three categories. In detail, the Majority Voting [9], Weighted Majority Voting [10], Behavior Knowledge Space [11], and Naive-Bayes Combination [12] methods are belong to the first category; the Class Set Reduction methods (including intersection of neighborhoods and union of neighborhoods) and the Class Set Reordering methods (including highest rank method, Borda count method and logistic regression method) [13] belong to the second category; the Class-Conscious methods [14] and the Class-Indifferent methods (such as Decision Template combiner [15] and Dempster-shafer combiner [16]) belong to the last category.

On the other hand, ultra-wideband (UWB) technology is attractive for its potential applications in Wireless Personal Area Networks (WPAN) [17,18,19]. It employs the short pulses (with bandwidths of several GHz) to transmit its information symbols at low power [20]. The advantages of UWB also stem from its ultra-wideband nature, such as good information hiding ability and less sensitivity to multipath fading [21,22]. To reinforce its multiple-access (MA) ability in the multiuser occasion, UWB can be combined with traditional spread-spectrum (SS) techniques, which were firstly proposed by Scholtz in [23], and with subsequent analyses in [24,25,26]. Among them, direct sequence UWB (DS-UWB) is an efficient MA scheme [21,27,28], where binary phase-shift keying (BPSK) can be employed and a large number of active users can share the same frequency bandwidth simultaneously, interfering with others [29]. (Please note that, another important spectrum sharing strategy is the cognitive radio technique. However, due to the space limitation, this technique will not be studied in this paper. The interested readers can refer to [30] for more information.) Therefore, the multiple access interference (MAI) exists and conduces to the performance aggravation of the conventional detector (CD) (also called the single-user matched filter). (For this reason, a lot of receiver solutions have been proposed in the literature. In [31], the multiple antenna diversity was employed in single-user UWB systems. In [32], a stop-and-go strategy based on energy detection in CD receiver was applied to alleviate excessive noise collection in single-user UWB clustered multipath channels. In [33], a zonal based Rake receiver was further proposed for multiuser UWB systems.) Besides these, the multiuser detection (MUD) technique that can eliminate or weaken the negative effects of MAI is studied in [34,35,36,37,38,39,40,41,42,43]. Verdu proposed the optimum multiuser detector (OMD) for code division multiple access (CDMA) systems [34], and it can achieve the optimal bit error rate (BER) performance [35] and the perfect near-far effect (NFE) resistant ability [36]. To DS-UWB systems, this OMD method was introduced by Yoon and Kohno [20]. However, the computational complexity of OMD growing exponentially with the number of active users makes it impractical to use [37].

Consequently, in order to make the tradeoff between performance and complexity, various sub-optimal MUD algorithms have been studied in literatures. In [38], a Reduced Complexity Maximum Likelihood (RCML) algorithm was proposed, but its practical use seems still impossible. In [39], a multiuser frequency-domain (FD) turbo detector combing FD turbo equalization schemes with soft interference cancelation was presented, whereas its BER performance is unsatisfactory. A multiuser detection method using a novel genetic algorithm base on complementary error function mutation (CEFM) was discussed for UWB systems in [40] and other swarm intelligence based MUD algorithms were studied in [41,42,43]. However, it is clear that a single multiuser detector cannot always be well suited for a particular occasion, particularly when the multipath or shadow fading happens. In [44,45], distributed signal detection was extended to the multiuser problem, then the jointly optimum criterion and the individually optimum criterion were proposed for cooperating receivers. Essentially, the performance gain obtained by this overall procedure is due to its inherent diversity. However, this distributed MUD scheme is still of high computational complexity, as well as OMD. Besides, more antennas and more communication loads are involved in this procedure.

In this paper, motivated by the concepts of decision-level fusion and distributed signal detection in [41,45], we firstly apply the decision-level fusion techniques into the field of MUD. Then two novel MUD algorithms, that is the chairman arbitrating decision-level fusion criterion (CA-DFC) based MUD algorithm and the veto logic decision-level fusion criterion (VL-DFC) based MUD algorithm, are proposed for DS-UWB systems. In both of these methods, a set of sub-optimal detectors (including the minimum mean square error (MMSE) detector, the decorrelating (DEC) detector and the successive interference cancellation (SIC) detector) are used, and the preliminary decisions from these detectors will be merged together by CA-DFC or VL-DFC in the fusion center. The numerical results demonstrate that these two proposed MUD algorithms both have the much better BER performance and NFE resistant ability than other sub-optimal MUD algorithms, and even close to OMD. Meanwhile, the computational complexity of these novel methods is significantly lower than that of OMD. Compared with CA-DFC, VL-DFC based MUD method can achieve a little superior performance at the expense of its higher complexity. So these two proposed algorithms have their different application ranges.

The remainder of this paper is organized as follows. In Section 2, some typical sub-optimal MUD algorithms are reviewed and the decision-level fusion based MUD system model is constructed for DS-UWB systems. In Section 3, the optimal decision-level fusion criterion (O-DFC) and its simplified form, which is the majority voting decision-level fusion criterion (MV-DFC), are discussed. On the problem analysis of O-DFC and MV-DFC, two novel MUD algorithms based on CA-DFC and VL-DFC are proposed respectively, in Section 4. In Section 5, simulation experiments that compare the performance of different MUD algorithms are made. Conclusions are in Section 6.

## 2. Problem Statement

### 2.1. Some Typical MUD Algorithms for DS-UWB Systems

Here, we let a *K*-user synchronous DS-UWB system under the additive white Gaussian noise (AWGN) channel, which could also be regarded as the results of the processing of rake receivers in the multipath channel. For this reason, we only give the multiuser DS-UWB model in the AWGN case, while it can be generalized to the multipath case easily.

In this multiuser DS-UWB system, each information symbol is spread over multiple pulses using pseudo random sequences (PRS). The BPSK modulation is employed by each user and the transmitted signal of the *k*th user can be expressed as:

(1) $$S_{tr}^{\left(k\right)}(t)=\sum_{j=-\infty }^{\infty }\sum_{n=0}^{N_{c}-1}b_{j}^{\left(k\right)}p_{n}^{\left(k\right)}w_{tr}(t-jT_{f}-nT_{c})$$

where {*b_j_^(k^*^)^} is the information symbols of the *k*th user, {*p_n_*^(k)^} denotes the PRS assigned to the *k*th user, *T_c_* is the pulse repetition period (namely the chip period), *T_f_* is the time duration of information symbol that satisfies *T_f_* = *N_c_T_c_*, and *N_c_* is the length of PRS. Besides, w*_tr_*(*t*) represents the transmitted pulse waveform that can be characterized as the second derivative of Gaussian pulse [20,21]:

(2) $$w_{tr}(t)=[1-4\pi (\frac{t}{\tau_{m}}{)}^{2}]\cdot \exp[-2\pi (\frac{t}{\tau_{m}}{)}^{2}]$$

where τ*_m_* is the parameter that controls the width of this pulse.

If these *K-* users are all active, the whole received signal of this system is

(3) $$r(t)=\sum_{k=1}^{K}A_{k}S_{tr}^{\left(k\right)}(t)+n(t)$$

where *A_k_* is the amplitude of the *k*th received signal and *n*(*t*) represents the received noise modeled as the AWGN, with a normal distribution *N*(0,σ_n_^2^).

According to [46], the output of a bank of single-user matched filters (MFs), called a conventional detector (*CD*), can be expressed in the following matrix and vector forms:

(4) y=RAb+ny

where *R* denotes the *K × K* normalized cross-correlation matrix with the *i*-*k*th entry $\rho_{ik}=\int_{0}^{T_{f}}S_{tr}^{\left(i\right)}(t)S_{tr}^{\left(k\right)}(t)dt$, *A* is a *K × K* diagonal matrix with the amplitude of the *k*th user’s received signal {*A_k_*}_*k=*1,2,…,*K*_ along the diagonal, b = (*b*_1_, *b*_2_, …, *b_K_*)*^T^* is the *K* × 1 vector containing the transmitted information bits from *K* users and the superscript “T” denotes a vector transpose operation. In addition, *n = (n*_1_, *n*_2_, …, *n_K_)*^T^ is the *K ×* 1 zero-mean Gaussian random noise vector with $n_{k}=\int_{0}^{T_{f}}n(t)S_{tr}^{\left(k\right)}(t)dt$ and its covariance matrix equal to

(5) $$E[nb^{T}]={\text{σ}}_{n}^{2}R$$

Due to the crude assumption that the MAI can be modeled as a zero-mean Gaussian random variable (called “Gaussian approximation”) for CD [47], its performance and multiuser capacity are limited. To improve this, Verdu proposed the optimum multiuser detection (OMD), which is based on the maximum likelihood (ML) criterion [35]. The objective function of OMD is given as:

(6) $$\hat{b}=\arg\;\underset{b\in {\left\{-1,+1\right\}}^{K}}{\max}(2b^{T}Ay-b^{T}ARAb)$$

From Equation (6), it is obvious that the selection of this optimal solution $\hat{b}$ in the *K*-dimensional Euclidean solution space is generally a Non-deterministic Polynomial (NP) hard problem [37]. That is, the computational complexity of OMD is *O* (2*^K^*), which grows exponentially with the number of active users. For this reason, despite its significant performance and capacity gains over CD, it is useless in practice. As a result, several sub-optimal MUD algorithms have been proposed to make the tradeoff between performance and complexity.

Linear MUD algorithm is one kind of sub-optimal approach, which processes the output of CD through multiplying by a matrix *M*. For example, in the decorrelating (DEC) detector, its transforming matrix is *M = R*^−1^; in the minimum mean square error (MMSE) detector, its transforming matrix is

(7) $$M=A^{-1}{\left[R+{{\text{σ}}_{n}}^{2}A^{-2}\right]}^{-1}$$

Moreover, as the linear detectors, such as CD, DEC, and MMSE detectors, the matrix multiplication is usually followed by a −1/+1 decision module:

(8) $$\hat{b}=\mathrm{sgn}(My)$$

where $\hat{b}$ is the preliminary decision vector.

Non-linear MUD algorithm is another kind of sub-optimal approach. For example, in the *successive interference cancellation (SIC) detector*, the decision of the *k*th user can be expressed as [46]:

(9) $${\hat{b}}_{k}=\mathrm{sgn}(y_{k}-\sum_{j=k+1}^{K}A_{j}{\text{ρ}}_{jk}{\hat{b}}_{j})$$

where the decision of the *i*th user, *i* = *k* + 1,*k* + 2, …, *K*, should be correctly demodulated, otherwise, its performance will be affected drastically. Thus, the order of demodulating users is the key problem for this detector. Here, we order users through Equation (10), which can be estimated easily from the outputs of CD:

(10) $$E[(\int_{0}^{T}r(t)S_{tr}^{\left(k\right)}(t)dt{)}^{2}]={\text{σ}}_{n}^{2}+{A_{k}}^{2}+\sum_{j\neq k}A_{j}^{2}{\text{ρ}}_{jk}^{2}$$

### 2.2. Decision-Level Fusion Based MUD System Model

**Figure 1:**
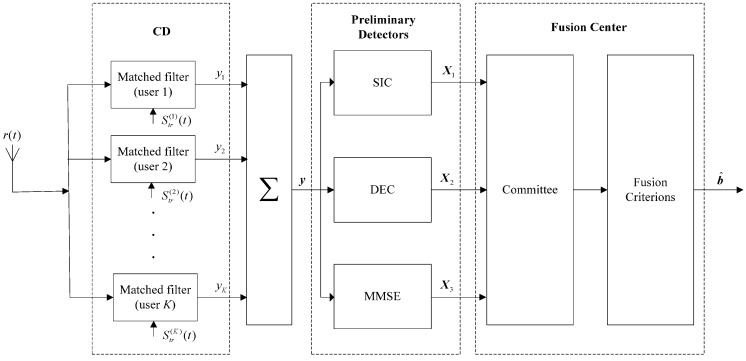
The structure of decision-level fusion based MUD system model.

In this paper, we consider the application of decision-level fusion techniques into the field of MUD. The decision-level fusion based MUD system model is constructed as Figure 1 shows. The characteristics of this scheme contain:

(1) The outputs of CD *y* = (*y*_1_, *y*_2_, …, *y_K_*)^T^ are passed to the preliminary sub-optimal detectors, including SIC, DEC, and MMSE detectors;
(2) The preliminary decisions of SIC, DEC, and MMSE (let *X*_1_, *X*_2_ and *X*_3_) are sent to the fusion center;
(3) The fusion center consists of two parts: committee and fusion criterions. Depending on different decision-level fusion criterions (DFCs), the committee can make a different final decision $\hat{b}$ on the basis of these preliminary decisions.
(4) This scheme is not distributed in fact, for it only needs one antenna to receive signals. Consequently, the computational complexity and apparatus integration are saved.

## 3. Optimal Decision-Level Fusion Criterion (O-DFC) and Its Simplified Form

**Figure 2:**
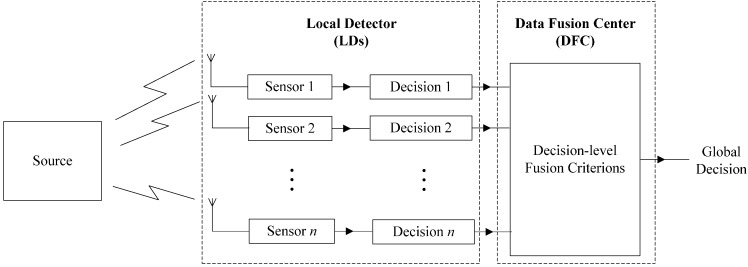
Distributed detection system with decision-level fusion center.

Since O-DFC has been discussed in the distributed detection system [48,49] (in Figure 2), here we simply review it and extend it to our decision-level fusion based MUD model (in Figure 1) in Section 3.1. Then the majority voting decision-level fusion criterion (MV-DFC), which is the simplified form of O-DFC, is given in Section 3.2.

### 3.1. Optimal Decision-Level Fusion Criterion (O-DFC)

In the distributed detection system as Figure 2 shows, signal processing is accomplished at the sensor and preliminary decisions are transmitted to the data fusion center. Then the global decision will be obtained as the output of this center, based on different DFCs. The optimal DFC (O-DFC) was derived by Chair and Varshney [48]. Here, we review it at first and then extend it to our model in Figure 1.

Assume a binary hypothesis testing problem, with two hypotheses:

(11) $$\left\{ \begin{array}{l} H_{0}:\text{ the signal is absent}\\ H_{1}:\text{ the signal is present} \end{array}\right.$$

where the priori probabilities of these hypotheses are *P*(*H*_0_) *= P*_0_ and *P*(*H*_1_) *= P*_1_. Consider there are n detectors in Figure 2 and the observations of each detector are denoted by *y_i_*, *i* = 1, 2, …, *n* with the assumption of statistical independence. In addition, the conditional probability density function is *p*(*y_i_|H_j_*), *i* = 1, 2, …, *n*, *j =* 0, 1.

As Figure 2 depicts, each detector can make its own decision *u_i_* by applying a decision rule g*_i_*(*y*_i_), *i* = 1, 2, …, *n*, where

(12) $$u_{i}=\left\{ \begin{array}{ll} -1, & \text{if }H_{0}\text{ is decided}\\ +1, & \text{if }H_{1}\text{ is decided} \end{array}\right.$$

These local preliminary decisions *u_i_* will be sent to the decision-level fusion center for the further processing. In general, the global decision is a function of these preliminary decisions as:

(13) $$u=f(u_{1},u_{2},\cdots ,u_{n})$$

According to [48], the minimum probability of error criterion can be employed to derive the O-DFC, which can be summarized as:

(14) $$u=f(u_{1},u_{2},\cdots ,u_{n})=\left\{ \begin{array}{ll} +1, & \text{if }a_{0}+\sum_{i=1}^{n}a_{i}u_{i}>0\\ -1, & \text{otherwise} \end{array}\right.$$

where the weights are given by

(15) $$a_{0}=\log\frac{P_{1}}{P_{0}}$$

(16) $$a_{i}=\left\{ \begin{array}{ll} \log\frac{1-P_{M_{i}}}{P_{F_{i}}}, & \text{if }u_{i}=+1\\ \log\frac{1-P_{F_{i}}}{P_{M_{i}}}, & \text{if }u_{i}=-1 \end{array}\right.$$

where *P_Fi_* = P(*u_i_* = +1|*H*_0_) is the false alarm probability and *P_Mi_* = *P*(*u_i_* = −1|*H*_1_) is the miss alarm probability of the *i*th detector, respectively. For more information of the detailed derivation, interested readers can refer to [48].

In our decision-level fusion based MUD model, however, these two hypotheses should be modified as:

(17) $$\left\{ \begin{array}{l} H_{0m}:-\text{1 is transmitted by the }m\text{th user}\\ H_{1m}:+\text{1 is transmitted by the }m\text{th user} \end{array}\right.$$

As a result, the false alarm probability P_Fim=P(u_im=+1|H_0m) and the miss alarm probability P_Mim=P(u_im=−1|H_1m) of the *i*th sub-optimal detector for the *m*th user are both equal to its bit error rate (BER) *P_eim_*, we have that is *P_Fim_ = P_Mim_ = P_eim_*, *i* = 1, 2, …, *n*, *m* = 1, 2, …, *K* and *K* is the number of users in this multiuser system. For the equiprobable source assumption, we have *a*_0_ = 0. Therefore, the O-DFC in our model can be rewritten by

(18) $$u_{m}=f(u_{1m},u_{2m},\cdots ,u_{nm})=\left\{ \begin{array}{ll} +1, & \text{if }\sum_{i=1}^{n}a_{im}u_{im}>0\\ -1, & \text{otherwise} \end{array}\right.$$

where *u_m_* is the final decision for the *m*th user, *u_im_* is the preliminary decision of the *i*th sub-optimal detector for the *m*th user, and

(19) $$a_{im}=\log\frac{1-P_{eim}}{P_{eim}}$$

In particular, for the special case when there are it {*n*} = 3 detectors, a proposition has been proposed by Chen *et al*. in [49]. Assuming the BER for the *m*th user at each detector be *P_eim_*, *i* = 1, 2, 3, this proposition reveals that the BER achieved at the fusion center , which is denoted as *P_ecm_O_*, is:

(20) $$P_{ecm\_O}=\min\{P_{e0m},P_{e1m},P_{e2m},P_{e3m}\}$$

where *P*_e0m_ = *P*_e1m_*P*_e2m_ + *P_e_*_1*m*_*P_e_*_3*m*_ *+ P_e_*_2*m*_*P_e_*_3*m*_ − 2*P_e_*_1*m*_*P_e_*_2*m*_*P_e_*_3*m*_. Therefore, from Equation (20), we can see that to achieve the improvement of performance by this fusion scheme, the following condition should be satisfied

(21) $$P_{e0m}<\min\{P_{e1m},P_{e2m},P_{e3m}\}$$

Otherwise, we should simply choose the detector having the lowest BER for the best performance, which also means that there is no performance gain in the fusion of these three detectors.

### 3.2. The Simplified form of O-DFC: the Majority Voting Decision-Level Fusion Criterion (MV-DFC)

To render the O-DFC tractable, we make a further assumption that for all sub-optimal detectors, their *P*_eim_ are all equal to *P*_em_, regardless of the differences between these detectors. That is, *a_im_* are all equal to *a_im_* in Equation (19). Besides, it is clear that the BER satisfies *P*_em_ < 0.5 (otherwise, the communication is meaningless), so that 1 − *P*_em_ > *P*_em_ and *a_m_* > 0.

Then we can derive the majority voting DFC, called MV-DFC, as follows:

(22) $$u_{m}=f(u_{1m},u_{2m},\cdots ,u_{nm})=\left\{ \begin{array}{ll} +1, & \text{if }\sum_{i=1}^{n}u_{im}>0\\ -1, & \text{otherwise} \end{array}\right.$$

For this case, the proposition obtained in [49] can be simplified to

(23) $$P_{ecm\_MV}=\min\{P_{e0m},P_{em}\}$$

where *P_e_*_0*m*_ = 3*P_em_*^2^ − 2*P_em_*^3^. So in order to improve the performance from *P_em_*, the following condition should be satisfied:

(24) $$P_{e0m}<P_{em}$$

That is,

(25) $$3P_{em}-2P_{em}^{2}<1$$

here we can get *P_em_* < 0.5 or *P_em_* > 1 from Equation (25). Nevertheless, consider *P_em_* is the BER conditioned on 0 < *P_em_* < 1, the condition 0 < *P_em_* < 0.5 should be satisfied to make an improvement. Obviously, it is quite easy to satisfy this condition in the normal communication systems.

## 4. Two Improved Decision-Level Fusion Criterions for MUD

### 4.1. The Problems in O-DFC and MV-DFC

Although two decision-level fusion criterions (O-DFC and MV-DFC) have been analyzed above, their performances are not acceptable in our decision-level fusion based MUD model, due to the following reasons:

(1) The optimal decision-level fusion performance that O-DFC can gain, is conditioned on the assumption that these local detectors are statistically independent with each other; meanwhile, the accurate BER value of each detector should be *a priori* knowledge. To our knowledge, these two assumed conditions are unsatisfied in fact.

**Figure 3:**
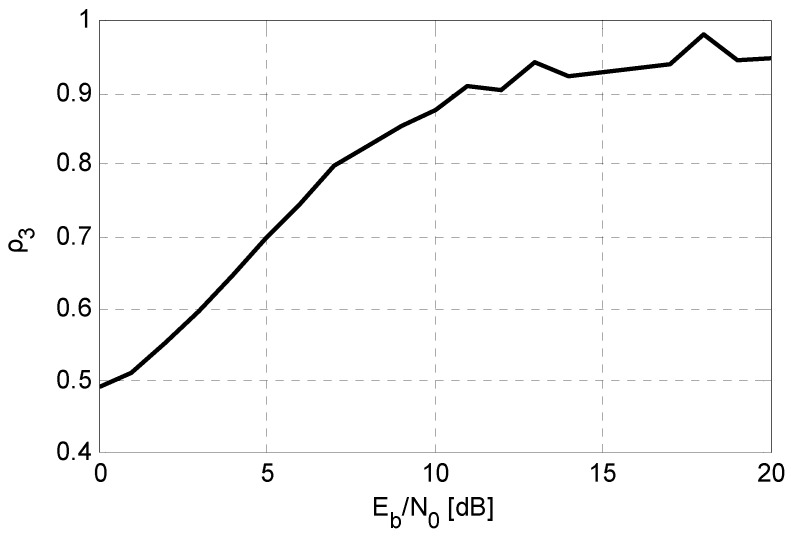
The correlation degree of SIC, DEC, and MMSE detectors, when *K* = 10.

On one hand, in Figure 1, the inputs of these sub-optimal detectors (SIC, DEC and MMSE) are all the outputs of CD. For this reason, their performances have a definite relationship with each other. Further, their correlation degree can be estimated [46] quantitatively:

(26) $${\text{ρ}}_{n}=\frac{nN^{f}}{N-N^{f}-N^{r}+nN^{f}}$$

where *n* is the total number of sub-optimal detectors, *N* is the total number of information bits for numerical tests, *N^f^* denotes the number of bits that are detected wrongly by all detectors while *N^r^* is the bits detected correctly by all. Figure 3 depicts the correlation degree ρ_3_ of SIC, DEC, and MMSE detectors *versus* *E_b_/N_0_*, when 10 active users are in this system and 106 information bits are used for this simulation. From this figure, we can see that as the *E_b_/N*_0_ increases, their correlation degree rises obviously until *E_b_/N*_0_ = 10 dB (from 0.48 to 0.87), while after that, it fluctuates slightly above 0.9.

On the other hand, since the communication channel is random, we are unlikely to estimate the BER of each detector accurately in practice.

(2) Compared with O-DFC, MV-DFC does not need the accurate estimation of the BER of each detector. Moreover, from Equation (22), only the operation of addition is demanded. However, its defects are also evident: (i) to make a final decision, the number of its sub-optimal detectors should be odd; (ii) the assumption of statistical independence is still necessary; (iii) even though the accurate estimation of BER is not needed, the condition *P_eim_* = *P_em_*, *i* = 1, 2, …, *n*, should still be satisfied, which is impossible for different sub-optimal detectors in our model.

From all the analysis above, we can conclude that both the O-DFC and the MV-DFC are not acceptable in our decision-level fusion based MUD model, and this will also be authenticated by numerical simulations in Section 5.

### 4.2. The Chairman Arbitrating Decision-Level Fusion Criterion (CA-DFC)

As Figure 1 shows, the preliminary decisions (let *X*_1_, *X*_2,_ and *X*_3_) of SIC, DEC, and MMSE detectors are transmitted to the committee synchronously. Note that it is a feature of the committee that there is a chairman in it, whose role is to evaluate these preliminary decisions and make the global decision [47]. The different ways to acquire this global decision correspond to the different DFCs. So here, we consider a situation where the chairman can make his arbitration among these sub-optimal or preliminary decisions with its own arbitrating rule, to select which one is the best. This method is called the chairman arbitrating DFC (CA-DFC) in this paper and discussed below.

Taking the objective function of OMD in Equation (6) into account, we define an approving degree function for the chairman to make his arbitration:

(27) $$D_{i}=2b^{T}Ay-X_{i}^{T}ARAX_{i}$$

where *i* = 1, 2, …, *n*. Then the CA-DFC can be derived:

If the term

(28) $$D_{j}=\underset{i=1,2,\cdots ,n}{\max}D_{i}$$

satisfies, the preliminary decision ***X**_j_* is arbitrated as the global decision by the chairman. In our model, *n* equals 3 for SIC, DEC, and MMSE detectors are employed, and this can be generalized into the case that has more than three detectors without difficulties.

Differing from O-DFC and MV-DFC, this CA-DFC doesn’t require the assumption of statistical independence between these sub-optimal detectors, as well as the accurate BERs of them. Furthermore, it can be speculated that the BER performance of this CA-DFC based MUD method is better than that of any of these sub-optimal detectors, due to the operation of Equation (28). That is, its BER achieves

(29) $$P_{ecm\_CA}<\min\{P_{e1m},P_{e2m},P_{e3m}\}$$

### 4.3. The Veto Logic Decision-Level Fusion Criterion (VL-DFC)

Another DFC introduced into the field of MUD in this paper is the veto logic (called VL-DFC). The veto logic implies that all sub-optimal detectors (all members) have to agree with the final decision that the chairman makes. If anyone of them dissents, the final decision should be abolished.

In this method, the voting bit is defined at first:

(30) $$u_{m}=f(u_{1m},u_{2m},\cdots ,u_{nm})=\left\{ \begin{array}{ll} +1, & \text{if }\sum_{i=1}^{n}u_{im}=+n\\ -1, & \text{if }\sum_{i=1}^{n}u_{im}=-n.\\ \times , & \text{otherwise} \end{array}\right.$$

where *m* = 1, 2, …, *K* and *K* is the number of users. Besides, the symbol “×” means this bit cannot be determined between +1 and −1 in this veto logic, while the term $\sum_{i=1}^{n}u_{im}=+n$ (or $\sum_{i=1}^{n}u_{im}=-n$) means that +1 (or −1) is the unanimous agreement reached by all sub-optimal detectors.

Through Equation (30), the *K*-dimensional voting vector is ***U*** = (*u*_1_, *u*_2_, …, *u_K_*)*^T^*, and the number of undetermined bits in it is *L (L < K).*

It is evident that there are 2^L^ likely solutions that the chairman should make a further decision. Here, we construct a simplified solution space, which consists of all these likely solutions, for the chairman to make his final decision. The approving degree function (Equation (27)) in CA-DFC is also applied to select the best one as the output of VL-DFC.

To summarize, this VL-DFC can be represented by the following steps:

(1) Calculate the *K*-dimensional voting vector ***U*** by Equation (30);
(2) Construct a simplified L-dimensional solution space based on ***U***, and L is the number of undetermined bits in ***U***;
(3) Compare the 2^L^ likely solutions in this space, and consider the solution that has the largest value of Equation (27) as the final decision made by the chairman.

Note that, the CA-DFC is a special case of VL-DFC, for the final decision in CA-DFC is bound to be involved in the simplified solution space of VL-DFC. Consequently, the BER performance of VL-DFC is better than that of CA-DFC, that is, *P_ecm_VL_* < *P_ecm_CA_*. Whereas, the performance improved by VL-DFC, compared with CA-DFC, is at the cost of its computational complexity increased. The detailed discussion about this is located in the following section.

## 5. Numerical Results and Analysis

In order to test and analyze these two proposed CA-DFC and VL-DFC based MUD algorithms (hereinafter called CA-DFC and VL-DFC, respectively), Monte Carlo simulations are utilized. As Figure 1 shows, a decision-level fusion based MUD receiver for DS-UWB systems is designed; meanwhile, in our experiments, the fusion center applies four DFCs: O-DFC, MV-DFC, CA-DFC, and VL-DFC. The major parameters used for these simulations are summarized in Table 1.

The performances of CD, SIC, DEC, MMSE, O-DFC, MV-DFC, CA-DFC, VL-DFC, and OMD are compared, including the BER performance *versus* *E_b_*/*N*_0_, the near-far effect (NFE) resistant capability, the BER performance *versus* the number of users *K*, and also their BER performance in the indoor multipath environment. Before them, the computational complexity of CA-DFC and VL-DFC is compared with that of OMD to demonstrate their efficiency.

**Table 1:** Simulation parameters.

System	DS-UWB
Modulation Mode	BPSK
Pseudo random sequences (PRS)	m sequences
The length of PRS	31
Communication channel	AWGN or IEEE 802.15.3a (CM2)
The number of testing information symbols	10^5^
The width of UWB pulse	0.7531 ns
The pulse repetition period	≈2 ns
The number of active users	*K* = 5, 10, 15, 20

### 5.1. The Computational Complexity Comparison

To compare the computational complexity of CA-DFC and VL-DFC with that of OMD, the total number of calculating the value of the objective function in Equation (6) per K × 1 information bit vector ***b*** is considered here (listed in Table 2). As the calculation of this function is not involved in CD, SIC, DEC, MMSE, O-DFC, and MV-DFC, the computational complexity of them is negligible.

**Table 2:** The computational complexity comparison.

MUD Algorithms	Calculation Number
CA-DFC	n
VL-DFC	2L
OMD	2K

**Figure 4:**
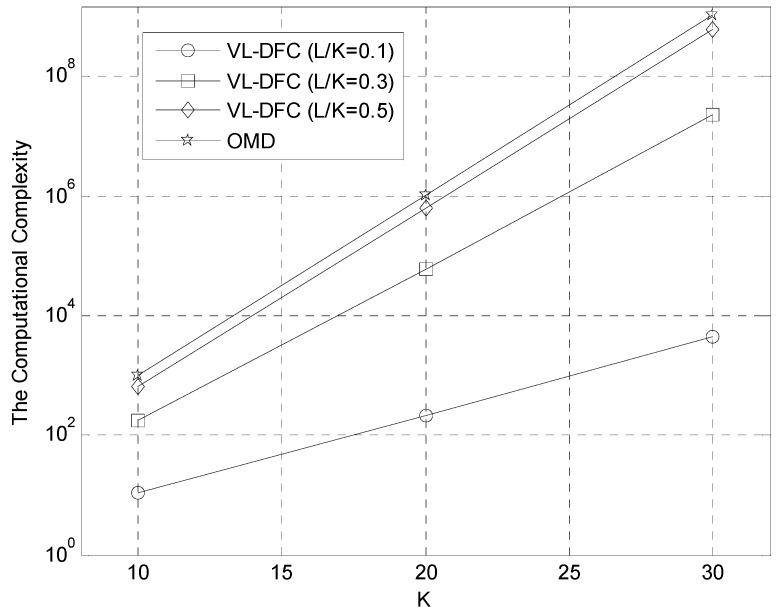
The computational complexity of VL-DFC and OMD.

In Table 2, we define *n* is the number of sub-optimal detectors used for fusion, and in this paper, *n* = 3. Besides, *L* is the number of undetermined bits in the voting vector, and *K* denotes the number of active users in this system. It is clear that the value of *L* has a relationship with *K*, and in general, the greater *K* is (and the stronger the MAI is), the greater *L* is. Therefore, the ratio *L/K* is adopted and then the computational complexity of VL-DFC and OMD is depicted in Figure 4, conditioned on *L/K* = 0.1, 0.3, 0.5, respectively.

**Figure 5:**
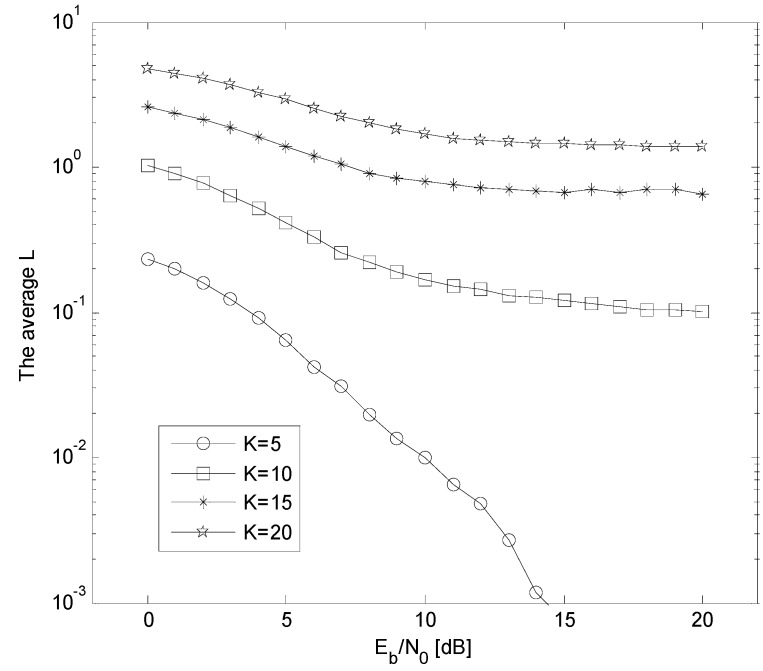
The average *L versus* *E_b_*/*N*_0_ curves when *K* = 5, 10, 15, and 20 in AWGN channel.

From the simulation results in Figure 4, we can see that as the number of users increases, the more computational complexity will be saved by VL-DFC, particularly when *L/K* is smaller than 0.3. In fact, this demand is satisfied as Figure 5 shows, which gives the average *L versus* *E_b_/N_0_* curves conditioned on *K* = 5, 10, 15 and 20 in AWGN channel. From this figure, three conclusions are obtained as follows: First, as *K* increases, the average *L* also increases obviously, which is due to the enhancement of MAI. Second, all the situations (points in this figure) can achieve *L/K* < 0.3. The last is that, as the *E_b_/N*_0_ is lower, the average *L* is greater, which means the solution space constructed in VL-DFC becomes larger for the chairman to make his global decision.

### 5.2. The BER Performance versus E_b_/N*_0_* Comparison

**Figure 6:**
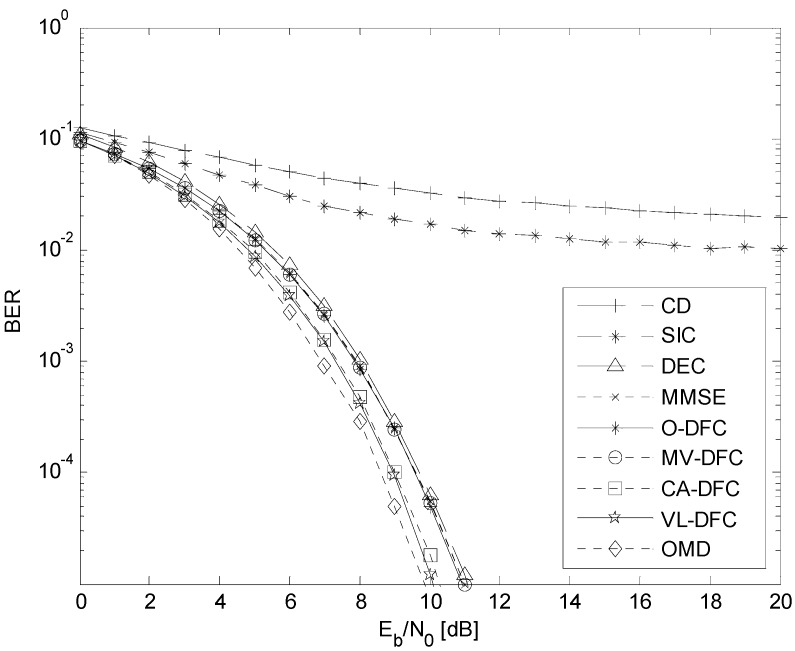
The BER *versus* *E_b_*/*N*_0_ curves in AWGN channel when *K* = 10 and perfect power control is employed.

The BER *versus* *E_b_/N*_0_ curves in the AWGN channel are depicted in Figure 6, when the number of users in this system is 10 and the energy per bit of all users is ensured to be equal by perfect power control, that is, *E_b_*_1_ *= E_b_*_2_ *= … = E_b_*_10_.

It can be seen from Figure 6 that the BER performances of these MUD algorithms can be divided into two types: one is the algorithms that the decision-level fusion is not considered, involving CD, SIC, DEC, MMSE, and OMD; the other is the algorithms that the decision-level fusion is considered on the basis of SIC, DEC, and MMSE as Figure 1 shows, involving O-DFC, MV-DFC, CA-DFC, and VL-DFC. In the first type, the BER performance of CD is the worst while that of OMD is the best, which has been demonstrated in the literature. However, due to the assumption of statistical independence between SIC, DEC, and MMSE is not satisfied, the BER performances of O-DFC and MV-DFC are even not better than that of MMSE. On the other hand, CA-DFC and VL-DFC both have much better BER performances than MMSE, O-DFC, and MV-DFC. Besides, compared with CA-DFC, VL-DFC is still a little better, which we have anticipated in Section 4.3.

Note that there is still a performance gap between VL-DFC (also CA-DFC) and OMD. To our knowledge, the reason is that in both our proposed methods, the situation when the preliminary detection results of these sub-optimal detectors (here, SIC, DEC, and MMSE) are all wrong is not considered. Since the assumption of statistical independence is not established, the estimation of this probability is out of this paper.

### 5.3. The NFE Resistant Ability Comparison

The BER performances of these algorithms without power control, named the near-far effect (NFE), are discussed in this simulation. In this DS-UWB system, there are also 10 users and the communication channel is AWGN.

**Figure 7:**
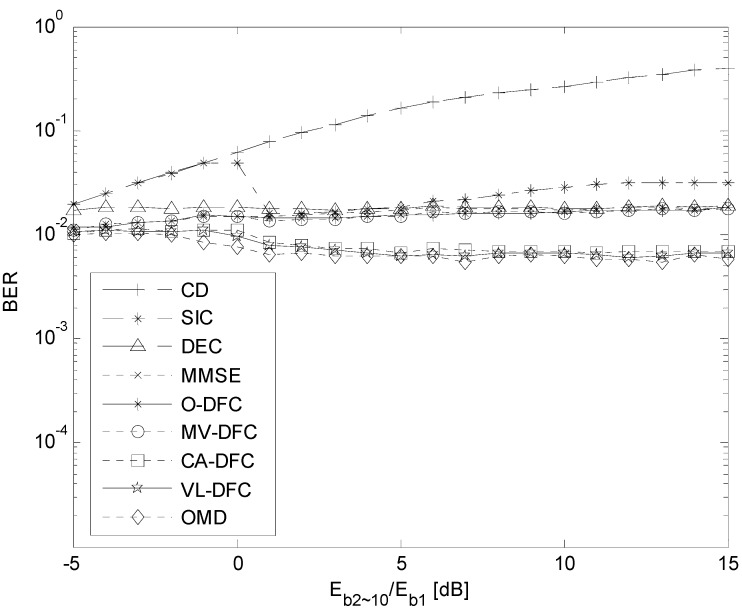
The BER *versus* *E_b_*_2~10_/*E_b_*_1_ curves in AWGN channel when *K* = 10 and no power control is employed.

Figure 7 shows the BER performance curves of the first user, when the energy per bit of this user is fixed with its *E_b_*_1_/*N*_0_ equal to 5 dB while that of other nine users *E_b_*_2~10_/*N*_0_ varies from 0 dB to 20 dB synchronously. That is, the ratio *E_b_*_2~10_/*E_b_*_1_ changes from −5 dB to 15 dB.

From this figure, we can see that the NFE resistant ability (no sense with *E_b_*_2~10_/*E_b_*_1_) of OMD is the best among them. Those of CA-DFC and VL-DFC are evidently much better than those of other sub-optimal detectors, and even close to that of OMD. Moreover, those of O-DFC and MV-DFC are still not better than that of MMSE for the same reason as well as Figure 6 shows.

Furthermore, the BER performance curve of SIC has an inflexion at the point where *E_b_*_2~10_/*E_b_*_1_ = 0 dB, due to its detection method in Equations (9) and (10). On one hand, when the energy per bit of users 2~10 calculated by Equation (10) is smaller than that of the first user, that is *E_b_*_2~10_/*N*_0_ < 5 dB and *E_b_*_2~10_/*E_b_*_1_ < 0 dB, then the information bits of user1 will be detected at first, the same as CD does. This is the reason the BER performance curve of SIC is identical with CD until *E_b_*_2~10_/*E_b_*_1_ = 0 dB. On the other hand, when *E_b_*_2~10_/*N*_0_ > 5 dB and *E_b_*_2~10_/*E_b_*_1_ > 0 dB, the information bits of users 2~10 will be detected before those of the first user, with more reliability. Consequently, after the interfering signal subtracted from the original received signal by Equation (9), the BER performance of SIC is improved dramatically.

### 5.4. The BER Performance versus K Comparison

**Figure 8:**
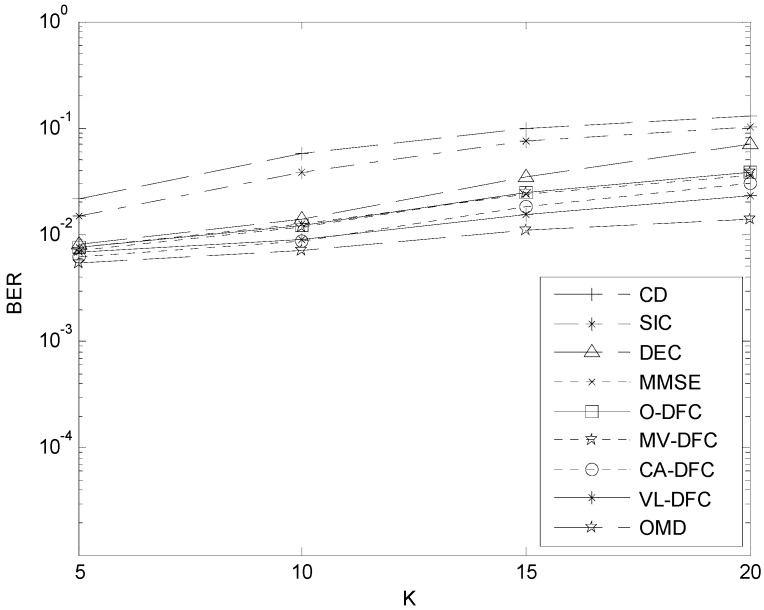
The BER *versus* *K* curves in AWGN channel when *E_b_*/*N*_0_ = 5 dB.

The BER performance curves of these detectors *versus* the number of active users *K* are displayed in Figure 8, when the energy per bit of all users is set *E_b_/N*_0_ = 5 dB. In general, as the number of users increases, the BER performances of all detectors become worse. In detail, OMD has the best capability to resist the effect of the increment of *K*, while CD has the worst. In addition, we can see that as *K* increases, the gap between VL-DFC and OMD enlarges. The reason for this phenomenon is that, the more users in this system, the more severely the MAI will be affected. So as *K* increases, the probability that the preliminary detection results of SIC, DEC, and MMSE are all wrong is bigger, resulting in the enlargement of this gap. But most importantly, the BER performances of CA-DFC and VL-DFC are both much better than other sub-optimal MUD algorithms. The achievement by introducing decision-level fusion techniques into MUD is significant.

### 5.5. The BER Performance Comparison in Multipath Environment

In order to verify the performances of CA-DFC and VL-DFC based MUD algorithms in the indoor environment, which is the most common situation where UWB technology can be employed, we carry out this experiment. Without loss of generality, the recommended IEEE 802.15.3a CM2 multipath channel [50,51] is used here, and the path with the strongest energy is gathered at the receiver.

**Figure 9:**
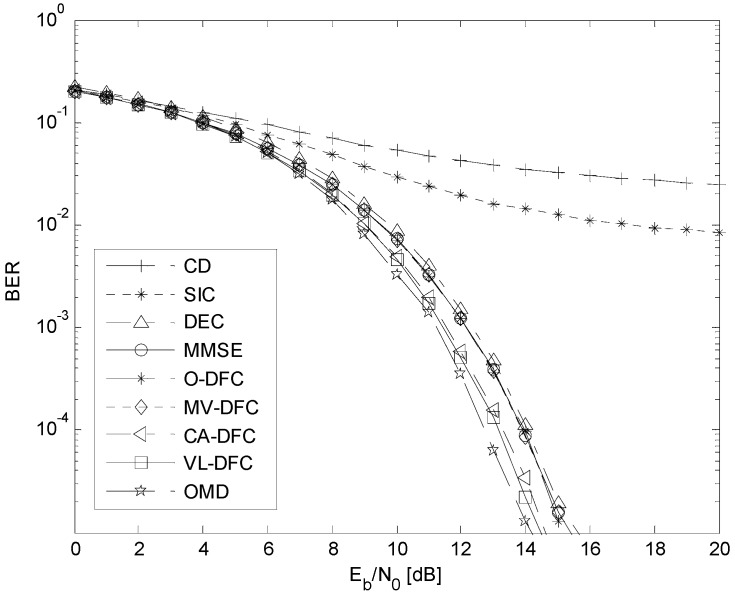
The BER *versus* *E_b_*/*N*_0_ curves in multipath channel when *K* = 10.

The BER performance curves of these MUD algorithms are compared in Figure 9. It is clear that the performances of these two proposed fusion-based algorithms are much better than those of other sub-optimal MUD algorithms, and even close to that of OMD. Taking account of the lower computational complexity they have, we can see that the good tradeoff between performance and complexity is realized by these novel fusion based algorithms.

## 6. Conclusions

In this paper, motivated by the concept of data fusion, we have proposed two novel decision-level fusion based MUD algorithms, called the CA-DFC based MUD algorithm and the VL-DFC based MUD algorithm. In the CA-DFC based scheme, the chairman at the fusion center can do his arbitration to select one of the preliminary decisions from sub-optimal MUD algorithms as the output of this scheme; while in the VL-DFC based scheme, the undetermined bits among these preliminary decisions are considered to construct a simplified solution space, and then the chairman should select the best solution within this space as the global decision. Computer simulations show that the BER performance and the NFE resistant ability of these two proposed algorithms are superior to those of other sub-optimal algorithms, and even close to those of OMD. Besides, the computational complexity of them is much lower than that of OMD, which indicates their practical use.
